# Endothelin-1 directs airway remodeling and hyper-reactivity in a murine asthma model

**DOI:** 10.1111/all.12271

**Published:** 2013-10-14

**Authors:** L G Gregory, C P Jones, S A Mathie, S Pegorier, C M Lloyd

**Affiliations:** Leukocyte Biology Section, National Heart and Lung Institute, Imperial CollegeLondon, UK

**Keywords:** animal models, asthma, epithelium, innate immunity, remodeling

## Abstract

**Background:**

The current paradigm describing asthma pathogenesis recognizes the central role of abnormal epithelial function in the generation and maintenance of the disease. However, the mechanisms responsible for the initiation of airway remodeling, which contributes to decreased lung function, remain elusive. Therefore, we aimed to determine the role of altered pulmonary gene expression in disease inception and identify proremodeling mediators.

**Methods:**

Using an adenoviral vector, we generated mice overexpressing smad2, a TGF-β and activin A signaling molecule, in the lung. Animals were exposed to intranasal ovalbumin (OVA) without systemic sensitization.

**Results:**

Control mice exposed to inhaled OVA showed no evidence of pulmonary inflammation, indices of remodeling, or airway hyper-reactivity. In contrast, local smad2 overexpression provoked airway hyper-reactivity in OVA-treated mice, concomitant with increased airway smooth muscle mass and peribronchial collagen deposition. Pulmonary eosinophilic inflammation was not evident, and there was no change in serum IgE or IgG1 levels. The profound remodeling changes were not mediated by classical pro-inflammatory Th2 cytokines. However, uric acid and interleukin-1β levels in the lung were increased. Epithelial-derived endothelin-1 and fibroblast growth factor were also augmented in smad2-expressing mice. Blocking endothelin-1 prevented these phenotypic changes.

**Conclusions:**

Innate epithelial-derived mediators are sufficient to drive airway hyper-reactivity and remodeling in response to environmental insults in the absence of overt Th2-type inflammation in a model of noneosinophilic, noninflammed types of asthma. Targeting potential asthma therapies to epithelial cell function and modulation of locally released mediators may represent an effective avenue for therapeutic design.

Asthma is a complex multifactorial inflammatory disease characterized by airway hyper-responsiveness (AHR) in response to a wide variety of inhaled environmental antigens. Structural changes to the airway wall collectively termed ‘airway remodeling’ contribute to airway dysfunction and, ultimately, provoke clinical symptoms. It has been proposed that the airway epithelium is central to the development of disease 1. Airway remodeling encompasses increased deposition of subepithelial extracellular matrix (ECM) proteins, increased smooth muscle mass, goblet cell hyperplasia, and angiogenesis 2. However, the relationship between inflammation and remodeling remains unclear, as remodeling occurs early in disease pathogenesis and is not affected by steroid therapy 3. Pharmacologically induced bronchoconstriction has been shown to promote airway remodeling in asthmatics without enhancing inflammation 4, implying that remodeling can be dissociated from inflammation. However, as all patients examined were atopic asthmatics, they have an inflammatory phenotype. In mice, adenoviral-mediated epithelial overexpression of smad2, a TGF-β and activin A signaling molecule, potentiates remodeling and AHR, without exacerbating the eosinophilia and Th2 inflammation in response to the common aeroallergen, house dust mite 5. Phosphorylated smad2 is increased following allergen challenge and is overexpressed in asthmatic patients, correlating with increased deposition of ECM 6,7. Moreover, the closely related gene smad3 has recently been identified as an asthma susceptibility gene in a large-scale genome-wide association study 8.

To dissect the effect of altered epithelial gene expression on the development of airway remodeling without the confounding influence of concurrent inflammation, we investigated the pulmonary response to inhaled ovalbumin (OVA), a commonly used surrogate allergen that normally promotes mucosal tolerance in mice if inhaled in the absence of systemic sensitization 9. OVA-exposed mice overexpressing epithelial smad2 secrete airway smooth muscle (ASM) mitogens, including endothelin-1, fibroblast growth factor (FGF), and the innate signaling molecules IL-1β and uric acid, resulting in AHR and airway remodeling in the absence of eosinophilic or Th2-type inflammation. These data suggest that a single alteration in the gene expression profile of the airway epithelium can completely alter the pulmonary response to a normally innocuous aeroallergen. Our observations give credence to the idea that mucosally derived mediators can drive airway remodeling and AHR independent of inflammation in the context of altered epithelial gene expression. Moreover, epithelial cells are firmly established as master regulators, which can dictate the threshold at which immune responses to inhaled stimuli are initiated.

## Methods

Female BALB/c mice (Charles River, Margate, UK), 6–8 weeks old received either 15 μg OVA (0.5 mg/ml) (Sigma-Aldrich, Dorset, UK) or 30 μl of vehicle, PBS, intranasally 3 days a week for up to 3 weeks. Selected groups received a first-generation replication-deficient adenovirus serotype 5 containing murine smad2 cDNA (AdS) (2 × 10^9^ viral pfu in 25 μl PBS) or a control mock vector (AdC) 2 days prior to commencing instillation of either OVA or PBS 5. In addition, mice received either endothelin antagonist PD 142893 (Sigma, Dorset, UK) or vehicle control (PBS) via injection prior (i.p.) to intranasal challenge with OVA or PBS. All experiments were performed in accordance with UK Home Office guidelines.

Airway responsiveness was determined by direct measurements of resistance (RI) and compliance (Cdyn) in anesthetized and tracheostomized mice 5. Selected groups received salbutamol (2 μg in PBS i.n., Sigma) 20 min prior to measurement of lung function and 24 h postfinal OVA challenge.

Serum, bronchoalveolar lavage (BAL), lung tissue, and lung cells were collected 5. Differential cell counts were carried out on Wright–Giemsa-stained cytospins. Paraffin-embedded sections (4 μm) were stained with hematoxylin/eosin (H&E), periodic acid-Schiff (PAS), and Sirus Red. All scoring and measurements were performed on medium airways measuring 150–250 μm in diameter. Paraffin sections were stained with rabbit anti-mouse proliferating cell nuclear antigen (PCNA) (Abcam, Cambridge, UK), α-smooth muscle actin (α-SMA) (Abcam), FGF-2 (Santa Cruz Biotechnology, Santa Cruz, CA, USA), P-smad2 (S465/467) (Cell Signaling Technology, Danvers, MA, USA), or endothelin (BiossInc, Woburn, CA, USA) using an avidin/biotin staining method. Recently synthesized acid-soluble collagen was measured in the lung by biochemical assay (Sircol collagen assay; Biocolor, Belfast, UK) and normalized for tissue weight.

Lung tissue supernatant was analyzed by ELISA using IL-4, IL-5, IFN-γ (PharMingen, Oxford, UK), IL-33, IL-25 (R&D systems, Abingdon, UK), IL-1α and IL-1β (active forms), IL-6 (eBioscience, Hatfield, UK), endothelin (Enzo Life Sciences, Exeter, UK), and IL-13 kits (R&D systems). Uric acid was measured using Amplex® red uric acid/uricase assay kit (Invitrogen, Paisley, UK). All data were normalized for lung weight. Paired antibodies for IgE and IgG1 (R&D systems) were used to measure serum Ig levels.

Disaggregated lung cells were stained with CD3, CD4, and ST2 or relevant isotype controls for 20 min at 4°C. Fixed cells were analyzed on a FACSCalibar™ using CellQuest (BD Biosciences, Oxford, UK).

Additional details on the methods utilized in this study are provided in the Supporting Information (Data S1).

Data were analyzed using Prism 4 (GraphPad software Inc., La Jolla, CA, USA). Multiple comparisons were performed using Kruskal–Wallis test. A two-tailed *P* value was determined by the Mann–Whitney *U*-test when comparing between two groups. Data shown represent means ± SEM of at least two independent experiments (*n *=* *6–12).

## Results

### Local smad2 overexpression promotes AHR in response to inhaled OVA in the absence of pulmonary inflammation

**Figure 1:**
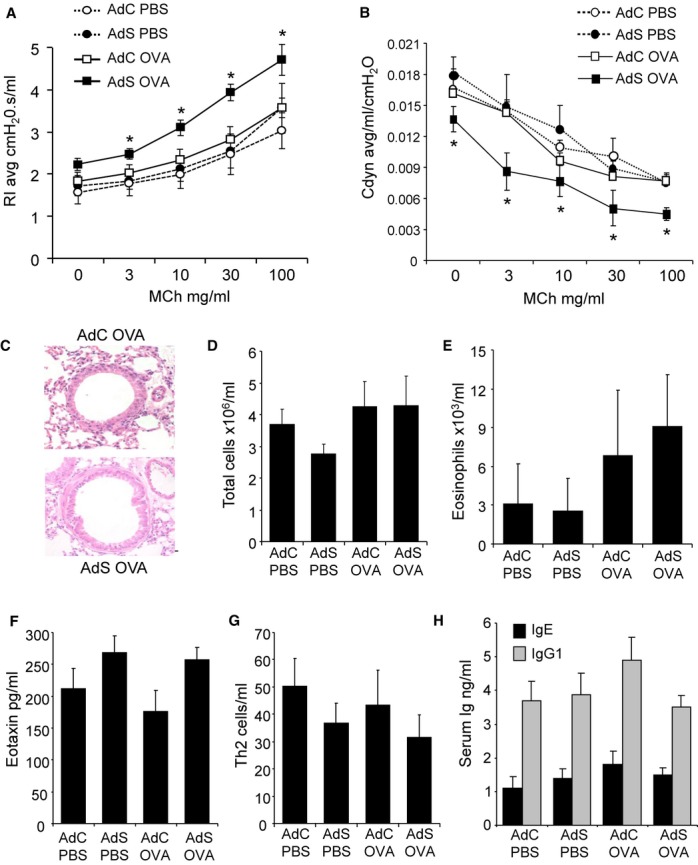
Ovalbumin (OVA) exposure results in airway hyper-responsiveness (AHR) in the absence of inflammation in mice overexpressing smad2 in the airway epithelium. (A) Resistance and (B) compliance measured in tracheotomized animals in response to increasing doses of methacholine. (C) Lung sections stained with H&E. Original magnification ×40. Scale bar = 50 μm. Representative photomicrographs are shown. (D) Total cells recovered from lung tissue. (E) Eosinophils determined by differential counting of cytospins prepared from the lung digest. (F) Eotaxin levels in the lung as determined by ELISA (assay sensitivity 3 pg/ml). (G) CD4^+^T1ST2^+^ Th2 cells recovered from the lung and quantified by flow cytometry. (H) Serum total IgE and IgG1 levels measured by ELISA (assay sensitivity 2 ng/ml and 80 pg/ml, respectively). Data shown represent means ± SEM (*N* = 6–18) of at least two independent experiments. *P < 0.05 compared with PBS-treated groups or OVA-exposed AdC mice.

Intranasal instillation of OVA in naïve BALB/c mice leads to immunological tolerance 9. In this study, we evaluated how altered expression of smad2 in the lung affects the pulmonary responses to inhaled OVA. We have shown previously that administration of the control adenoviral vector (AdC) or virus containing the transgene smad2 (AdS) results in gene expression localized primarily in epithelial cells of the conducting airways and does not itself initiate an inflammatory response in the lung 5. Therefore, in all experiments described here, AdC was used as an appropriate control. Overexpression of smad2 had no effect on baseline AHR. However, AdS mice exposed to OVA showed significantly increased airway resistance and decreased compliance in response to methacholine when compared with PBS-treated mice or the AdC OVA group (Fig. 1A,B). Asthma is often characterized by eosinophilic inflammation. However, there was no evidence of pulmonary inflammation in any of the groups of mice (Fig. 1C). Quantitatively there were no significant differences in the total number of leukocytes or eosinophils recovered from the lung (Fig. 1D,E) or BAL fluid (data not shown) in mice exposed to intranasal OVA compared with PBS. Similarly, levels of eotaxin were comparable between all groups (Fig. 1F). There was no difference in Th2 cell numbers in the OVA-treated mice compared with PBS controls (Fig. 1G). Likewise, serum total IgE and IgG1 were not modulated (Fig. 1H). OVA-specific IgE and IgG1 were also not detectable (data not shown). Mucosal OVA-specific IgA levels were slightly increased in the BALF of OVA-treated mice; however, there was no difference comparing AdC OVA with AdS OVA mice (data not shown). Thus, altered epithelial smad2 expression influences the development of AHR after OVA challenge, but does not initiate an overt pulmonary inflammatory reaction.

### Inhaled OVA exposure activates airway epithelial cells and induces collagen deposition in mice overexpressing smad2

**Figure 2:**
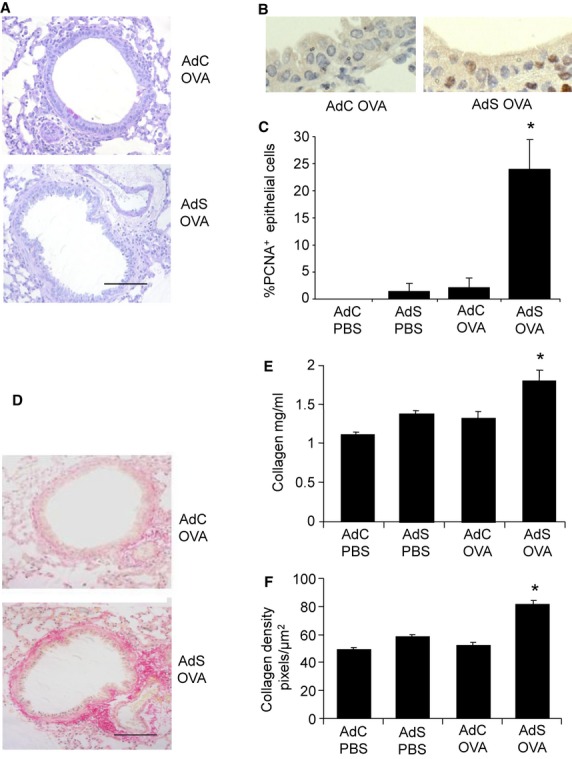
Airway remodeling induced by ovalbumin (OVA) in epithelial smad2-overexpressing mice. (A) Lung sections stained with periodic acid-Schiff (PAS) to identify mucin-containing (purple) cells. (B) Lung sections stained with antibody to proliferating cell nuclear antigen (PCNA, brown staining). (C) Quantitation of PCNA^+^ airway epithelial cells. (D) Sirius Red staining of lung sections depicts peribronchiolar and perivascular collagen (red). (E) Recently synthesized total lung collagen was quantified by a biochemical (Sircol) assay. (F) Quantitative image analysis of subepithelial peribronchiolar collagen density determined by measuring Sirius Red-stained collagen in lung sections under polarized light. Original magnification ×40. Scale bar = 50 μm. Representative photomicrographs are shown. Data shown represent means ± SEM (*N* = 6–12) of two independent experiments. *P < 0.05 compared with PBS-treated groups or OVA-exposed AdC mice.

Epithelial PAS staining for mucous was absent from lungs of all treatment groups (Fig. 2A), indicating there was no change to a mucous-secreting phenotype. Interestingly, however, there was an increase in the number of PCNA^+^ epithelial cells (Fig. 2B,C). PCNA is expressed by S-phase-proliferating cells suggesting that despite the absence of mucus hyperplasia, the airway epithelium of the AdS OVA-exposed mice is in an activated state.

PBS-treated mice demonstrated minimal collagen staining around the airways. In contrast, mice overexpressing smad2 and exposed to OVA showed a notable increase in the deposition of collagen compared with PBS-treated or the AdC OVA mice (Fig. 2D). Recently synthesized total lung collagen was increased in the AdS OVA mice (Fig. 2E), reflecting the specific increase in peribronchiolar collagen density (Fig. 2F).

### Inhaled OVA exposure increases ASM mass and contractility in AdS mice

**Figure 3:**
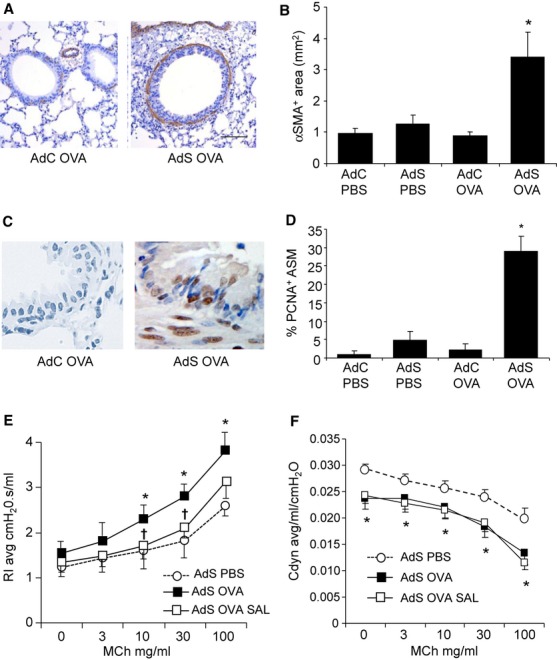
Airway smooth muscle changes induced by ovalbumin (OVA) in AdS mice. (A) Lung sections stained with an antibody against α-smooth muscle actin (α-SMA, brown staining), depicting myofibroblasts and smooth muscle cells. (B) Quantification of α-SMA^+^ peribronchiolar area. (C) Lung sections stained with an antibody against proliferating cell nuclear antigen (PCNA, brown staining). (D) Quantitation of PCNA^+^ peribronchiolar mesenchymal cells. Original magnification ×40. Scale bar = 50 μm. Representative photomicrographs are shown. (E) Airway resistance and (F) compliance measured in tracheotomized animals in response to increasing doses of methacholine. Data shown represent means ± SEM (*N* = 4–12) of two independent experiments. *P < 0.05 compared with PBS-treated groups or OVA-exposed AdC mice. †*P* < 0.05 comparing AdS OVA-challenged mice treated with salbutamol or vehicle, PBS.

Lung sections were immunostained with α-SMA to identify myofibroblasts and smooth muscle cells. Discontinuous α-SMA^+^ cells were observed in the PBS-treated groups and AdC OVA mice. In contrast, a continuous layer of peribronchiolar α-SMA^+^ cells was quantified in the AdS OVA mice (Fig. 3A,B). The extent of ASM hyperplasia was determined by calculating the percentage of PCNA^+^ subepithelial cells. This index of cellular proliferation was increased in the AdS OVA group compared with PBS controls and AdC OVA-treated mice (Fig. 3C,D).

The β2-adrenoceptor agonist, salbutamol, blocked the increase in airway resistance in the OVA-exposed mice overexpressing smad2 (Fig. 3E), demonstrating a pivotal role of smooth muscle contraction in the alteration of lung function. In contrast, the decreased airway compliance was not reversed (Fig. 3F). To confirm that the observed proremodeling effects in the AdS OVA mice were not solely due to contamination of the OVA preparation by bacterial-derived endotoxins, we utilized endograde® OVA, which contains <1 EU LPS/mg. An identical phenotype was observed in the AdS endograde® OVA mice as the AdS OVA mice (Fig. S1).

### Mediators of airway remodeling

We measured levels of the pro-inflammatory cytokines IL-1α, IL-6, and IL-33; Th2 cytokines IL-4, IL-5, IL-13, and IL-25; the Th1 cytokine IFN-γ; and the pleiotropic growth factor TGF-β in the lung and BALF. However, no differences in the levels of any of the mediators tested were observed between groups at either week 1, prior to the onset of remodeling changes and development of AHR, or at week 3 when AHR and remodeling are established (Table S1).

### Innate epithelial mediators are increased in AdS OVA mice

**Figure 4:**
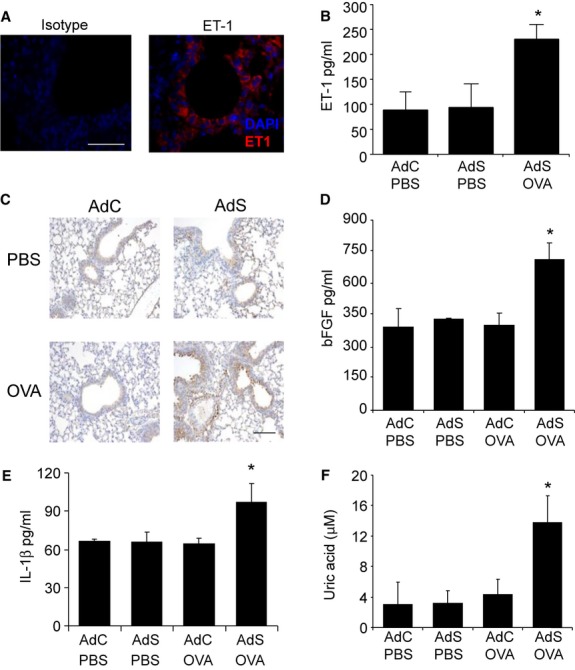
Ovalbumin (OVA) increases mediators of airway remodeling and airway hyper-responsiveness (AHR) in mice overexpressing smad2 in the airway epithelium. (A) Endothelin-1 expression (red-stained) in airway epithelial cells correlates with (B) endothelin-1 levels in the BALF (assay sensitivity 0.41 pg/ml). (C) Lung sections stained with an antibody against fibroblast growth factor (FGF, brown staining). (D) Pulmonary FGF-2 levels. (E) Pulmonary IL-1β (assay sensitivity 8 pg/ml) and (F) uric acid levels (assay sensitivity 100 nM). Representative photomicrographs are shown. Original magnification ×40. Scale bar = 50 μm. Data shown represent means ± SEM (*N* = 6–18) of at least two independent experiments. *P < 0.05 compared with PBS-treated groups or OVA-exposed AdC mice.

To investigate the mechanism by which altered pulmonary gene expression affects AHR and remodeling, we measured levels of mediators associated with innate immune signaling. Endothelin-1 is a potent bronchoconstrictor peptide with growth-promoting properties 10. Endothelin-1 is produced by airway epithelial cells (Fig. 4A), and increased levels of this mediator are released into the airways of AdS OVA mice (Fig. 4B). Increased expression of epithelial-derived FGF was also observed in these mice (Fig. 4C,D). Pulmonary levels of IL-1β (Fig. 4E) and the damage-associated molecular pattern, uric acid (Fig. 4F), were also significantly increased. Increased levels of these mediators were detected as early as 1 week following initial OVA challenge (Fig. S2).

### Blocking endothelin abrogates airway remodeling and AHR

**Figure 5:**
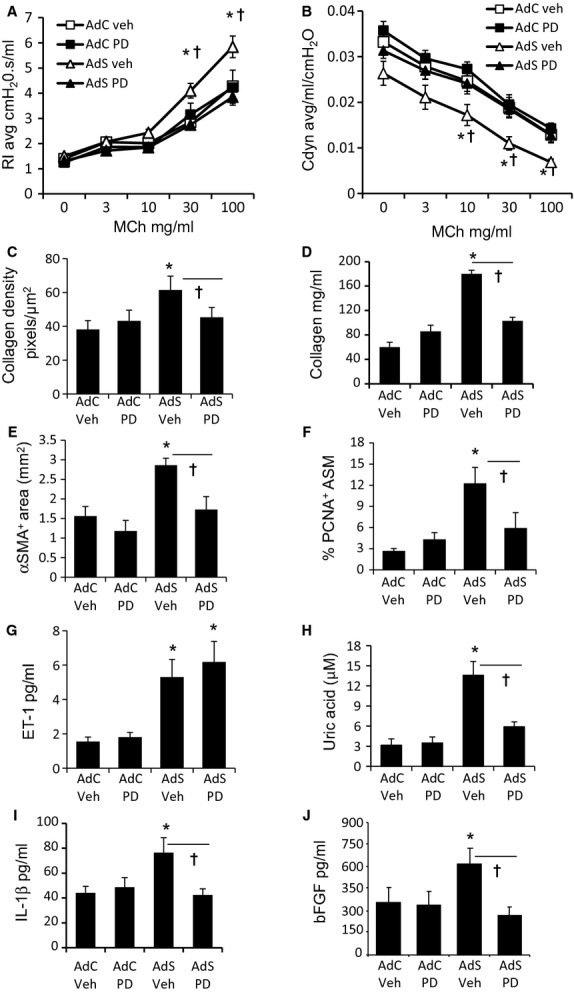
Airway remodeling and airway hyper-responsiveness (AHR) are driven by endothelin-1 and uric acid. (A) Resistance and (B) compliance measured in tracheotomised animals in response to increasing doses of methacholine. (C) Quantitative image analysis of subepithelial peribronchiolar collagen density determined by measuring Sirius Red-stained collagen in lung sections under polarized light. (D) Recently synthesized total lung collagen was quantified by a biochemical (Sircol) assay. (E) Quantification of α-smooth muscle actin (α-SMA)^+^ peribronchiolar area. (F) Quantitation of proliferating cell nuclear antigen (PCNA)^+^ peribronchiolar mesenchymal cells. (G) Pulmonary endothelin-1, (H) uric acid, (I) IL-1β, and (J) fibroblast growth factor-2 (FGF-2) levels. Data shown represent means ± SEM (*N* = 6–12) of two independent experiments. *P < 0.05 compared with PBS-treated groups or ovalbumin (OVA)-exposed AdC mice. †*P* < 0.05 comparing AdS OVA-challenged mice treated with PD142893 to AdS OVA PBS.

To determine whether endothelin has a role in driving the observed airway remodeling, AdS OVA mice were treated with the nonselective endothelin receptor antagonist PD 142893 (Fig. 5). Endothelin receptor blockade prevented the OVA-induced AHR (Fig. 5A,B), associated collagen deposition (Fig. 5C,D) and ASM hyperplasia (Fig. 5E,F). Although pulmonary endothelin levels were unaffected by antagonism of its receptor (Fig. 5G), the downstream effectors, uric acid, IL-1β, and FGF, were reduced to baseline in the PD 142893-treated AdS OVA mice (Fig. 5H–J).

## Discussion

Airway remodeling is a recognized feature of the asthmatic lung, and the epithelium is a prominent source of ECM components and fibrogenic mediators. However, the mechanisms responsible for remodeling and the relative contribution of the bronchial epithelium in this process are still poorly understood. Local smad2 overexpression resulted in enhanced AHR and airway remodeling in response to intranasal OVA challenge. Despite the pronounced effects on lung function and remodeling, there was no evidence of eosinophilia or increased Th2 cells within the lung. Additionally, there was no change in the levels of Th2-associated cytokines, and no effect on B-cell responses as serum levels of IgE and IgG1 were not increased in AdS mice compared with AdC control groups irrespective of OVA challenge. Instead AHR and remodeling was associated with the specific induction of epithelial-derived smooth muscle mitogens.

Asthma is heterogeneous with respect to clinical features 11,12, and Th2-type inflammation is characteristic only in a proportion of patients. Paucigranulocytic asthma is a phenotype with little or no inflammation based on the cellular composition of induced sputum samples. Multivariate cluster approaches to phenotyping asthma have also identified a distinct group of female asthmatics with a preponderance of obesity and lack of eosinophilia 11. Additionally, airway remodeling in pediatric severe therapy-resistant asthma has recently been shown not to correlate with Th2 cytokines 13.

Animal models allow dissection of mechanistic pathways, which may be important in driving disease pathology. Although the model presented here does not represent a model of clinical disease, it provides a vehicle for the study of remodeling in the absence of underlying inflammation. Using this approach, we show unequivocally that AHR and remodeling driven by altered pulmonary gene expression can develop independently of eosinophilia or T-cell recruitment, which is impossible to do in human subjects. In the context of altered gene expression, exposure to a usually innocuous stimulus results in epithelial-derived innate mediator release, which promotes airway remodeling and AHR. Intrinsic abnormalities of epithelial cells, ASM contractility, or remodeling may provide a mechanism for noninflammatory/Th2-low asthma.

Pretreatment of mice with salbutamol abrogates the increased airway resistance in AdS OVA-challenged mice, suggesting that the enhanced resistance is mainly due to increased contractility of smooth muscle. In contrast, the decrease in pulmonary compliance is likely due to the increased peribronchiolar collagen deposition, and the conducting airways remain ‘stiff’ due to excess ECM, which has previously been associated with a decline in lung function in asthmatic patients and the loss of bronchodilator reversibility 10,14. Treatment of AdS OVA mice with the endothelin antagonist prevents the increase in peribronchiolar collagen deposition and associated reduction in airway compliance.

Endothelins promote synthesis of ECM proteins by epithelial cells and fibroblasts, myofibroblast differentiation, and proliferation of mesenchymal cells 15,16. They have also been implicated in the generation of AHR 17,18. Levels of endothelin-1 were increased in AdS OVA-treated mice and correlate with the observed increase in peribronchiolar ASM mass and collagen deposition. Increased levels of endothelin-1 are also detected in the BAL fluid and serum from severe asthmatics, particularly those with steroid refractory asthma, and correlate with increased ASM area 19,20. Endothelin receptors are expressed on fibroblasts and myofibroblasts 21, and endothelin-1 can both promote fibrogenesis and decrease myofibroblast susceptibility to apoptosis 22. FGF has been shown to directly stimulate proliferation and migration of ASM cells *in vitro* 23, and there is data to support its role as an ASM mitogen in human asthma 24. Endothelin-1 has previously been shown to increase FGF-2 expression *in vitro* 25, and here we show increased pulmonary FGF-2 in AdS OVA mice. Endothelin receptor blockade prevented the AHR and airway remodeling in AdS OVA mice. These data suggest that activation of airway epithelium leads to secretion of endothelin-1 and FGF-2 with subsequent proliferation of ASM cells and secretion of ECM.

Airway remodeling and AHR correlated with increases in pulmonary IL-1β, a pro-inflammatory cytokine involved in the pathogenesis of asthma that can induce AHR 25,26. Asthmatic epithelia grown at air–liquid interface secrete more basolateral IL-1β following epithelial injury than cells derived from nonasthmatics 27. *In vitro* studies using human corneal and intestinal epithelial cell monolayers have shown that IL-1β decreases transepithelial resistance and increases permeability resulting in redistribution of tight junction proteins 28,29. Similar perturbations of the airway epithelium have been suggested to drive the genesis of asthma 30. IL-1β is produced by cleavage of an inactive pro-IL-1β precursor by autocleavage of caspase-1, a protease activated by NLRP3 inflammasomes 31. Human airway epithelial cells respond to particulate matter by increasing NLRP3 inflammasome-mediated IL-1β production 32. Intracellular NLRP3 senses endogenous danger signals such as uric acid, resulting in inflammasome activation 33. Hyperuricemia is associated with myocardial hypertrophy and remodeling mediated via an endothelin-1 pathway 34. The current data suggest that a similar uric acid–endothelin-1 axis may operate in the lung to promote fibrosis as increased uric acid levels were observed in the lungs of AdS OVA mice. The levels of endothelin-1, uric acid, and IL-1β were increased as early as 7 days following the first OVA challenge, prior to the onset of remodeling changes. This is perhaps not surprising as innate mediator release from epithelial cells does not require elements of the adaptive immune system and epithelial cells respond rapidly to ‘danger’ signals, particularly in the AdS OVA mice, which have an altered epithelial phenotype.

It may seem surprising that there was no observable neutrophilia despite increases in IL-1β and uric acid. Although significant, the increase in both of these mediators was relatively small compared with levels previously reported in other models of allergic airway diseases 35. It is plausible that there is a threshold for inflammatory mediators, which must be reached to promote a systemic inflammatory response and recruitment of cells from the blood and bone marrow. In contrast, small increases in endothelin-induced IL-1β and uric acid acting locally at the mucosal surface, at least in association with altered pulmonary gene expression, are sufficient to promote extreme changes in the bronchial architecture where the threshold for a remodeling response is lowered.

Although the exact mechanism by which OVA induces pathophysiology in the Ads OVA mice remains enigmatic, other examples of non-receptor-mediated signaling have been described. Hypoxia increases TGF-β signaling and subsequently endothelin-1 resulting in pulmonary remodeling 36. Similarly, inhaled asbestos and silica also cause lung damage without binding to receptors on pulmonary cells. Pertinent to our study, the downstream effects of both these environmental insults are mediated via IL-1β 37. Conceivably instilled OVA may be endocytosed by airway epithelial cells, as demonstrated in cultured 16HBE cells. We have demonstrated an increase in smad2 phosphorylation only in OVA-exposed mice overexpressing epithelial smad2 (Fig. S3). In mice with perturbed epithelial gene expression, this is sufficient stimulus to induce innate smad-mediated increases in endothelin-1 synthesis and subsequently the initiation of remodeling changes.

Thus, even relatively innocuous antigens such as OVA can trigger innate immune pathways and promote profound remodeling changes in the lung in the context of susceptible airway epithelium. These data consolidate the central role of the epithelium in determining the response of the lung to environmental airway challenge. Successful development of therapeutics to target mucosally derived mediators would likely be of particular benefit to patients with noneosinophilic, noninflammed types of asthma where current treatments, aimed at modulating inflammation and IgE, are ineffective at modulating AHR and airway remodeling.

## Supporting Information

Figure S1 Airway remodeling and AHR is not dependent on LPS contamination.

Figure S2 Innate mediators are increased 1 week after first challenge in AdS OVA mice.

Figure S3 Phosphorylation of smad2 in AdS OVA mice.

Table S1 Proinflammatory cytokines are not elevated in OVA treated mice.

Data S1 Mucosal derived innate signaling molecules direct airway remodeling and hyperreactivity.
