# Supplementary figures and images for: Endothelin-1 directs airway remodeling and hyper-reactivity in a murine asthma model
Source: Allergy. 2013 Oct 14;68(12):1579–88. doi: 10.1111/all.12271 (PMC3992903; doi:10.1111/all.12271)

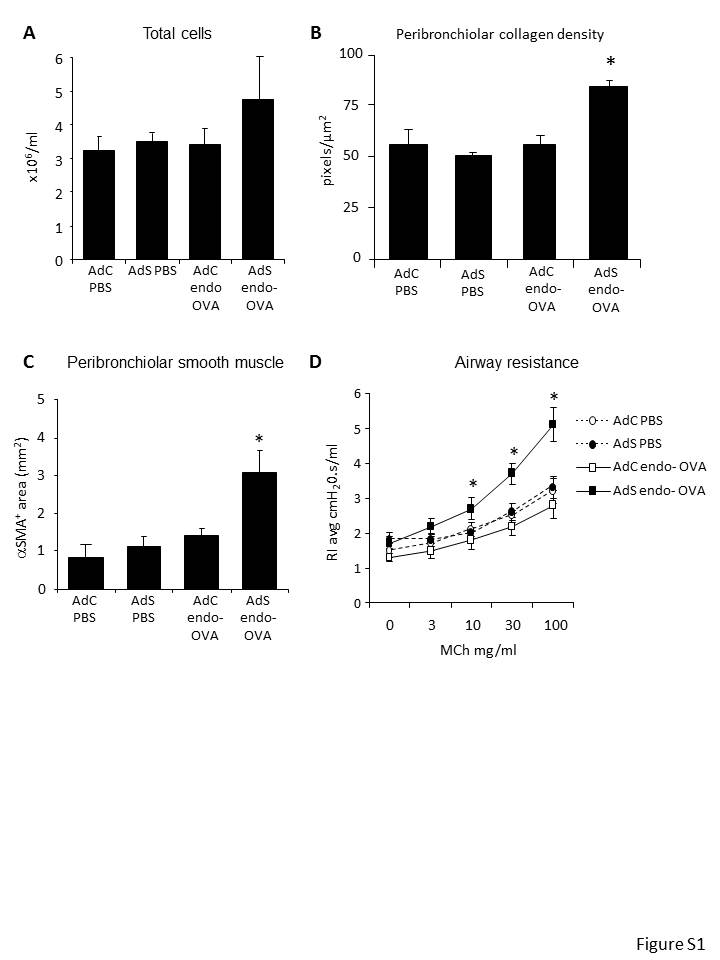

Supplement: Figure S1 — Airway remodeling and AHR is not dependent on LPS contamination. [file all0068-1579-sd1.jpg]

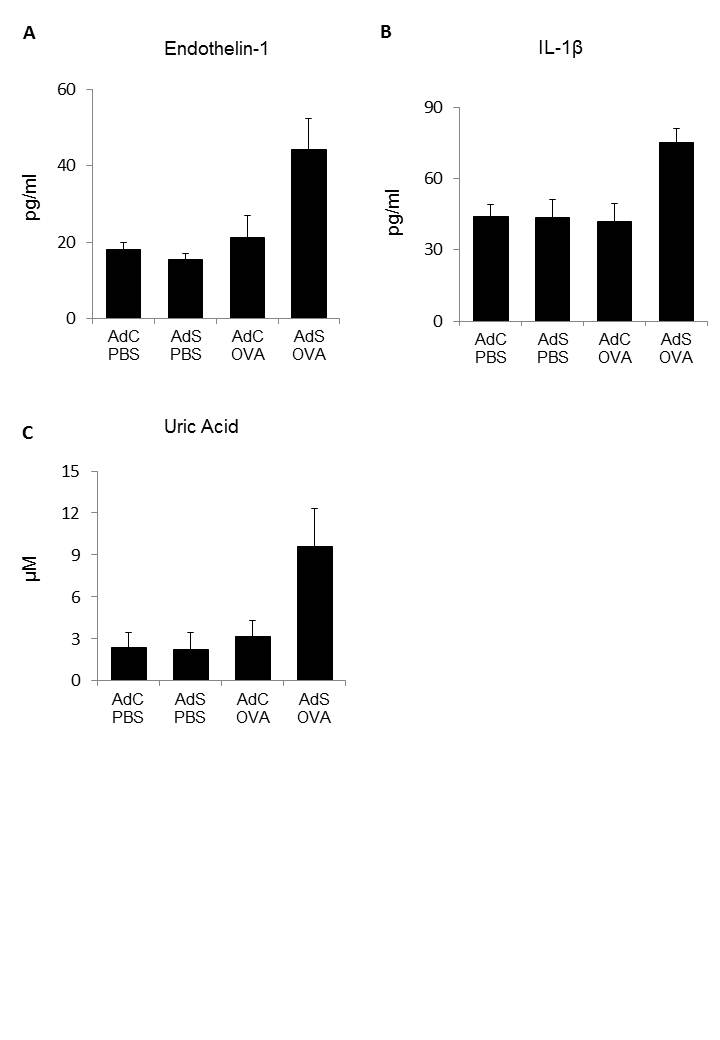

Supplement: Figure S2 — Innate mediators are increased 1 week after first challenge in AdS OVA mice. [file all0068-1579-sd2.jpg]

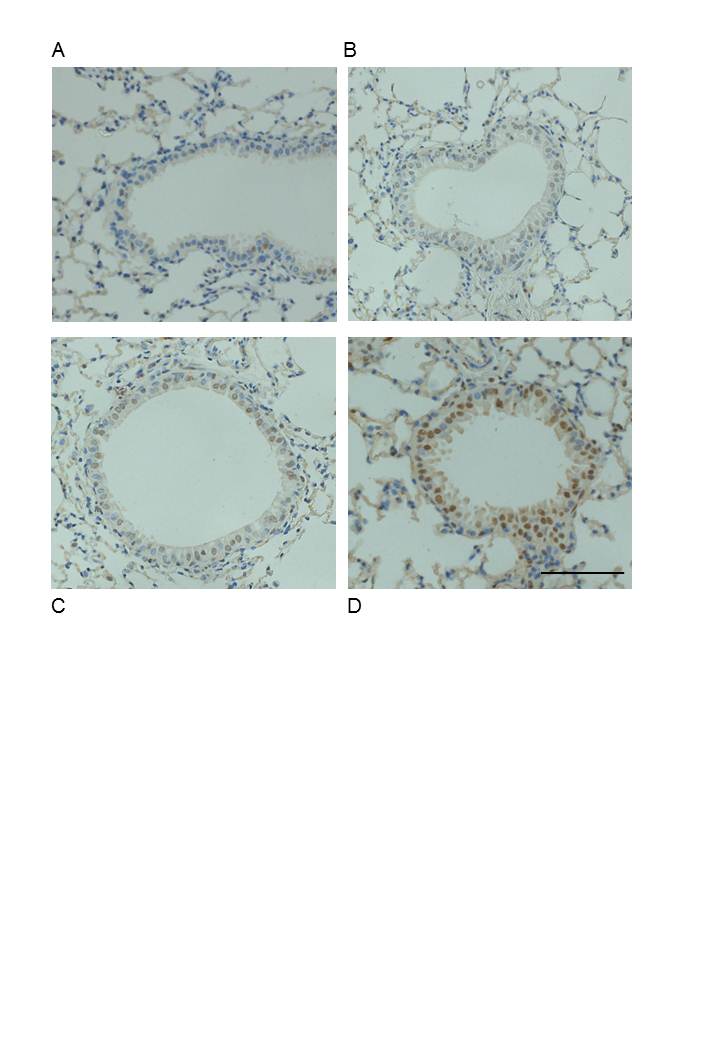

Supplement: Figure S3 — Phosphorylation of smad2 in AdS OVA mice. [file all0068-1579-sd3.jpg]

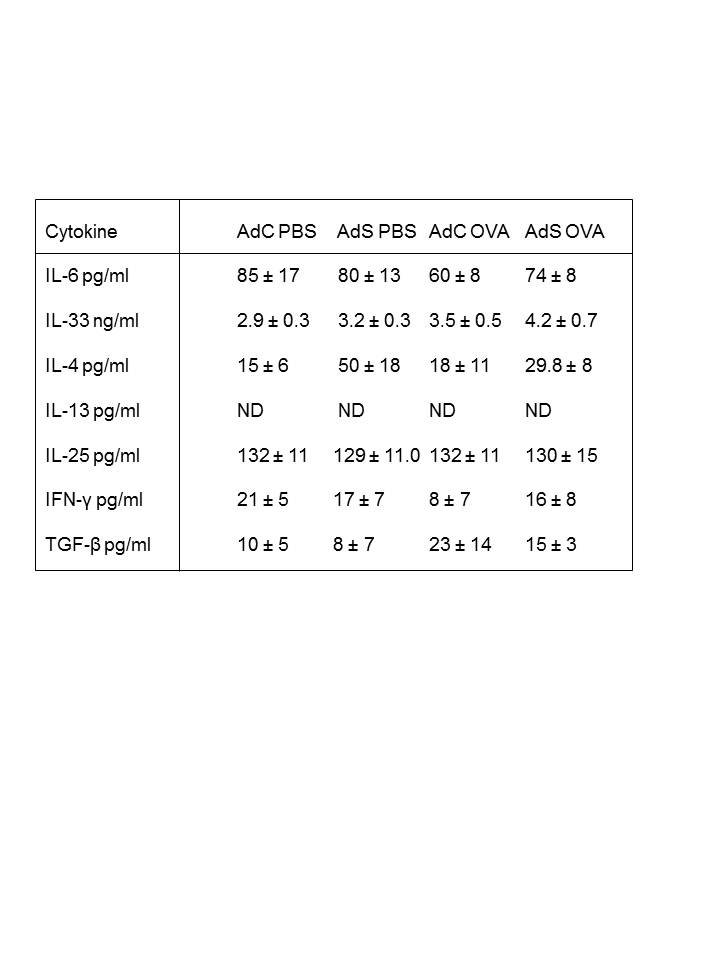

Supplement: Table S1 — Proinflammatory cytokines are not elevated in OVA treated mice. [file all0068-1579-sd4.jpg]
