# Supplementary material for: Endothelin-1 directs airway remodeling and hyper-reactivity in a murine asthma model
Source: Allergy. 2013 Oct 14;68(12):1579–88. doi: 10.1111/all.12271 (PMC3992903; doi:10.1111/all.12271)
Supplement: Data S1 — Mucosal derived innate signaling molecules direct airway remodeling and hyperreactivity. [file all0068-1579-sd5.docx]

Supplementary data

Mucosal derived innate signaling molecules direct airway remodeling and hyperreactivity

Lisa G. Gregory, Carla P. Jones, Sara A. Mathie, Sophie Pegorier & Clare M. Lloyd

Materials and Methods

**Measurement of AHR**

Direct measurements of dynamic lung resistance (R*_L_*) and pulmonary compliance (C*_dyn_*) were measured in anaesthetised and trachestomised mice.  Mice were ventilated using a small animal ventilator (Harvard Apparatus, Kent, UK) and measurements were taken in response to inhalational doses of the cholinergic agonist methacholine (MCh - Sigma, Poole, UK) at increasing concentrations (3mg/ml - 100mg/ml) in an EMMS system (Electro-Medical Measurement Systems, Bordon, Hants, UK) in a modified version of previously described methods[E1]. The trachea was exposed and cannulated then mice were ventilated at 150 breaths/minute with a stroke volume of 7ml/kg, comparable with normal breathing.  Baseline measurements were obtained for 2 mins followed by a PBS dose.  Each subsequent MCh dose lasted 20 seconds, and the response to each dose was measured for 4 minutes with measurements taken every 25 breaths.  Lung R*_L_* and C*_dyn_* values were averaged for each 4 minute period following MCh challenge and expressed as cmH_2_O/mls/s and ml/cmH_2_O respectively.

**Collection and preparation of samples**

Bronchoalveolar lavage (BAL) was collected by lavage of the lungs three times with 0.4 mL of PBS via a tracheal cannula. BAL fluid was centrifuged (700 *g*, 5 min, 4°C) and cells were recovered. BAL cell supernatants were removed and analyzed for cytokines by ELISA. To disaggregate the cells from the lung tissue, one lobe (
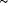
100 mg) of lung was incubated at 37°C for 1 h in digest reagent (0.15 mg/ml collagenase type D, 25 µg/ml DNase type I) in complete RPMI media. The recovered cells were filtered through a 70-µm nylon sieve, washed twice, resuspended in complete media, and counted in a hemocytometer prior to cytocentrifugation. Lung and BAL cells were applied to glass slides by centrifugation (5x10^4^ cells/slide) and stained with Wright-Giemsa (Thermo Fisher Scientific Inc, Waltham, MA). Percentages of macrophages, lymphocytes/mononuclear cells, eosinophils and neutrophils were determined under 40x magnification by counting cells in 8 randomly selected fields and dividing this number by the total number of cells counted. To obtain absolute numbers, this percentage was multiplied by the total number of cells recovered in 1 ml of lavage fluid and lung digest suspension which were normalised for the weight of the lung.

**Assessment of airway remodeling**

Goblet cells were visualized on periodic acid-Schiff (PAS)-stained lung sections and scored with results expressed as a mucus score in arbitrary units.[E2,3] PAS-stained goblet cells in airway epithelium were measured double-blind using a numerical scoring system (0: <5% goblet cells; 1: 5 to 25%; 2: 25 to 50%; 3: 50 to 75%; 4: >75%) The sum of airway scores from each lung was divided by the number of airways examined, 20–50 airways per mouse, and expressed as mucus cell score in arbitrary units.

Smooth muscle cell proliferation was assessed in lung sections stained with an antibody against PCNA. The percentage of PCNA^+^ airway mesenchymal cells (an index of smooth muscle hyperplasia) was calculated by counting the number of PCNA positive mesenchymal cells as a percentage of the total number of peribronchiolar mesenchymal cells.

Peribronchiolar collagen deposition was determined in Sirius red stained sections using Scion Image Analysis software package (Scion Corporation, Frederick, MD, USA) adapted from the literature.[E4] Digital photographs of four bronchioles per tissue section were taken at ×40 magnification under polarised light and these images were converted into monochrome. Ten measurements of 20 μm lines from each of the four bronchioles were drawn at a right angle from the basement membrane into the submucosa and the mean density of staining intensity along the 20 μm was calculated and expressed as pixels per μm^2^.

**Figure S1. Airway remodeling and AHR is not dependent on LPS contamination.** (A) Total cells recovered from the lung tissue. (B) Quantitative image analysis of subepithelial peribronchiolar collagen density determined by measuring sirius red stained collagen in lung sections under polarized light. (C) Quantification of α-SMA^+^ peribronchiolar area. (D) Resistance measured in tracheotomised animals in response to increasing doses of methacholine. Data shown represent means ± SEM (N=4-8). * = p < 0.05 compared with with PBS treated groups or endograde® OVA exposed AdC mice.

**Figure S2. Innate mediators are increased 1 week after first challenge in AdS OVA mice.** (A) endothelin-1 levels in the BALF (assay sensitivity 0.41 pg/ml). (B) IL-1β (assay sensitivity 8 pg/ml) and (C) uric acid levels (assay sensitivity 100nM). Data shown represent means ± SEM (N=4-8).

**Figure S3. Phosphorylation of smad2 in AdS OVA mice.** Immunohistochemistry demonstrating psmad2 expression (brown staining) in (A) AdC PBS, (B) AdS PBS, (C) AdC OVA and (D) AdS OVA mice. Representative photomicrographs are shown. Original magnification x 40. Scale bar = 50µm.

Table S3. Proinflammatory cytokines are not elevated in OVA treated mice. Cytokine levels measured in lung homogenate following 3 weeks OVA exposure. Data shown are means ± SEM (N=8-12).

**References**

E1. Martin TR, Gerard NP, Galli SJ, Drazen JM.  Pulmonary responses to bronchoconstrictor agonists in the mouse. *J Appl Physiol* 1988;64:2318–2323.

E2. Grunig G, Warnock M, Wakil AE,  [Venkayya R](http://www.ncbi.nlm.nih.gov/pubmed?term=Venkayya%20R%5BAuthor%5D&cauthor=true&cauthor_uid=9856950), [Brombacher F](http://www.ncbi.nlm.nih.gov/pubmed?term=Brombacher%20F%5BAuthor%5D&cauthor=true&cauthor_uid=9856950), [Rennick DM](http://www.ncbi.nlm.nih.gov/pubmed?term=Rennick%20DM%5BAuthor%5D&cauthor=true&cauthor_uid=9856950), [Sheppard D](http://www.ncbi.nlm.nih.gov/pubmed?term=Sheppard%20D%5BAuthor%5D&cauthor=true&cauthor_uid=9856950), [Mohrs M](http://www.ncbi.nlm.nih.gov/pubmed?term=Mohrs%20M%5BAuthor%5D&cauthor=true&cauthor_uid=9856950), [Donaldson DD](http://www.ncbi.nlm.nih.gov/pubmed?term=Donaldson%20DD%5BAuthor%5D&cauthor=true&cauthor_uid=9856950), [Locksley RM](http://www.ncbi.nlm.nih.gov/pubmed?term=Locksley%20RM%5BAuthor%5D&cauthor=true&cauthor_uid=9856950),  [Corry DB](http://www.ncbi.nlm.nih.gov/pubmed?term=Corry%20DB%5BAuthor%5D&cauthor=true&cauthor_uid=9856950). Requirement for IL-13 independently of IL-4 in experimental asthma. *Science* 1998;282:2261-2263.

E3. Townsend JM, Fallon GP, Matthews JD,  [Smith P](http://www.ncbi.nlm.nih.gov/pubmed?term=Smith%20P%5BAuthor%5D&cauthor=true&cauthor_uid=11070175), [Jolin EH](http://www.ncbi.nlm.nih.gov/pubmed?term=Jolin%20EH%5BAuthor%5D&cauthor=true&cauthor_uid=11070175), [McKenzie NA](http://www.ncbi.nlm.nih.gov/pubmed?term=McKenzie%20NA%5BAuthor%5D&cauthor=true&cauthor_uid=11070175). IL-9-deficient mice establish fundamental roles for IL-9 in pulmonary mastocytosis and goblet cell hyperplasia but not T cell development. *Immunity* 2000;13:573-583.

E4. Flood-Page P, Menzies-Gow A, Phipps S, Ying S, Wangoo A, Ludwig MS, Barnes N, Robinson D, Kay AB. Anti-IL-5 treatment reduces deposition of ECM proteins in the bronchial subepithelial basement membrane of mild atopic asthmatics. *J Clin Invest* 2003;112:1029–36.
